# Supplementary material for: Inter-subspecies diversity of maize to drought stress with physio-biochemical, enzymatic and molecular responses
Source: PeerJ. 2024 Aug 22;12:e17931. doi: 10.7717/peerj.17931 (PMC11345000; doi:10.7717/peerj.17931)
Supplement: Supplemental Information 1 [file peerj-12-17931-s001.docx]

Suppl. Table 1. Mean squares obtained from variance analysis of examined parameters for the effect of in maize subspecies in drought stress.

| Source | DF | RWC | LOT | PRO | SPR | MD% | MDI | MDA | H_2_O_2_ | SOD | APX | CAT | HSP70 | HSP90 |
| --- | --- | --- | --- | --- | --- | --- | --- | --- | --- | --- | --- | --- | --- | --- |
| Maize subspecies (M) | 2 | 231.4^**^ | 201.11^**^ | 249.68^**^ | 66186^**^ | 96.77^*^ | 42.92^*^ | 22.79^**^ | 1507.67^**^ | 0.02^**^ | 0.46^**^ | 0.05^**^ | 3.44^**^ | 134.30^**^ |
| Drought stress (D) | 2 | 2844.0^**^ | 2273.11^**^ | 173.33^**^ | 901878^**^ | 312.64^**^ | 199.55^**^ | 76.81^**^ | 341.75^**^ | 0.06^**^ | 0.43^**^ | 0.40^**^ | 0.11^ns^ | 83.28^**^ |
| M × D | 4 | 676.5^**^ | 550.46^**^ | 323.84^**^ | 25507^**^ | 52.33^ns^ | 34.69^ns^ | 12.21^**^ | 11.74^*^ | 0.01^ns^ | 0.10^**^ | 0.02^**^ | 1.02^ns^ | 97.86^**^ |
| Error | 18 | 10.8 | 13.71 | 0.93 | 2063 | 23.13 | 14.88 | 2.71 | 4.28 | 0.003 | 0.004 | 0.001 | 0.54 | 12.46 |

^*^ = p ≤ 0.05, ^**^ = p ≤ 0.001, ^ns^ = not significant, RWC: relative water content, LOT: loss of turgidity, SPR: soluble protein content, MDA: malondealtehit content, H2O2: hydrogen peroxide content, SOD: superoxide dismutase activity, APX: ascorbate peroxidase activity, CAT: catalase activity, HSP70: HSP70 gene expression level, HSP90: HSP90 gene expression level
