# Supplementary figures and images for: Inter-subspecies diversity of maize to drought stress with physio-biochemical, enzymatic and molecular responses
Source: PeerJ. 2024 Aug 22;12:e17931. doi: 10.7717/peerj.17931 (PMC11345000; doi:10.7717/peerj.17931)

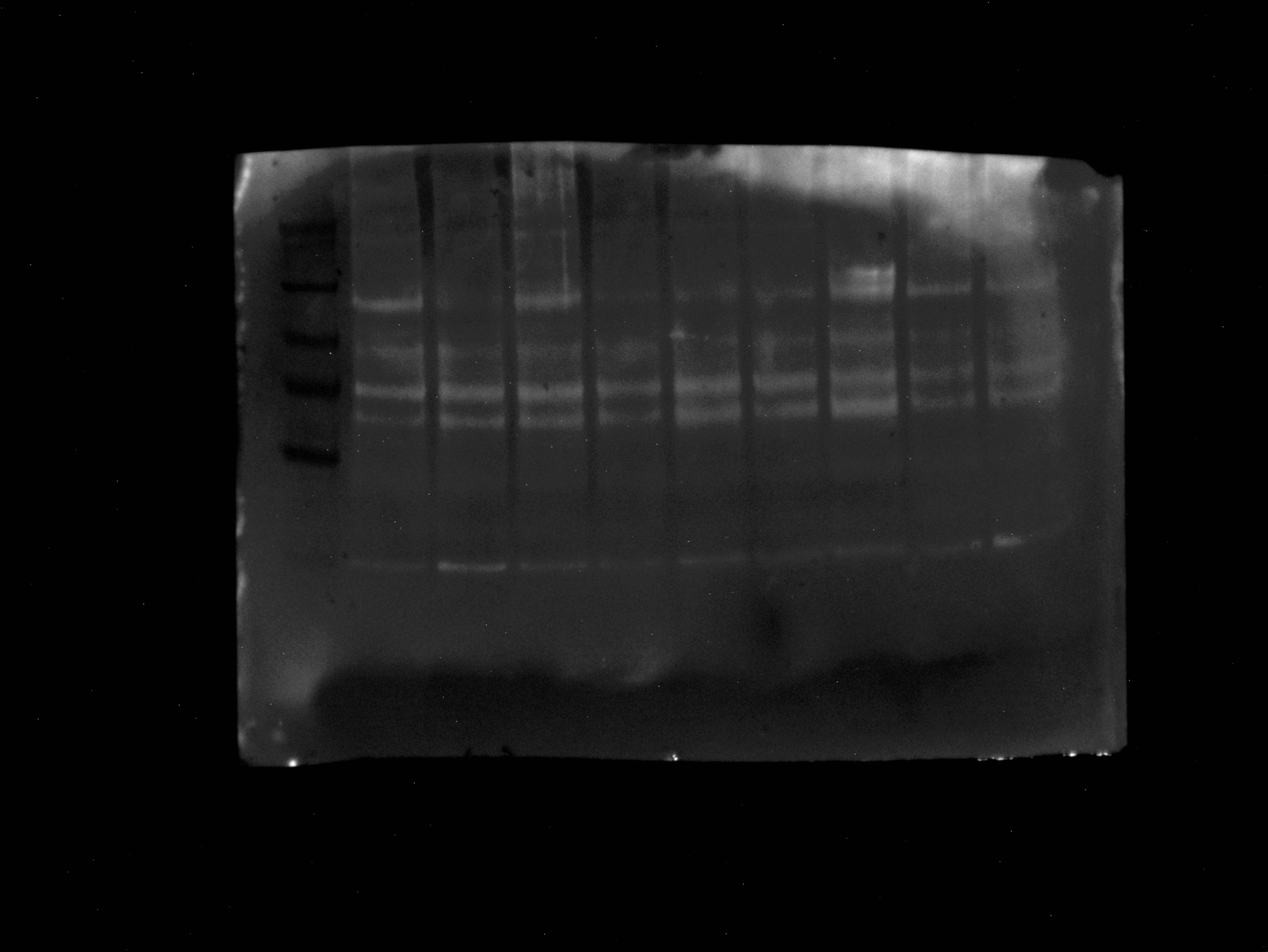

Supplement: Supplemental Information 4 [file peerj-12-17931-s004.jpeg]

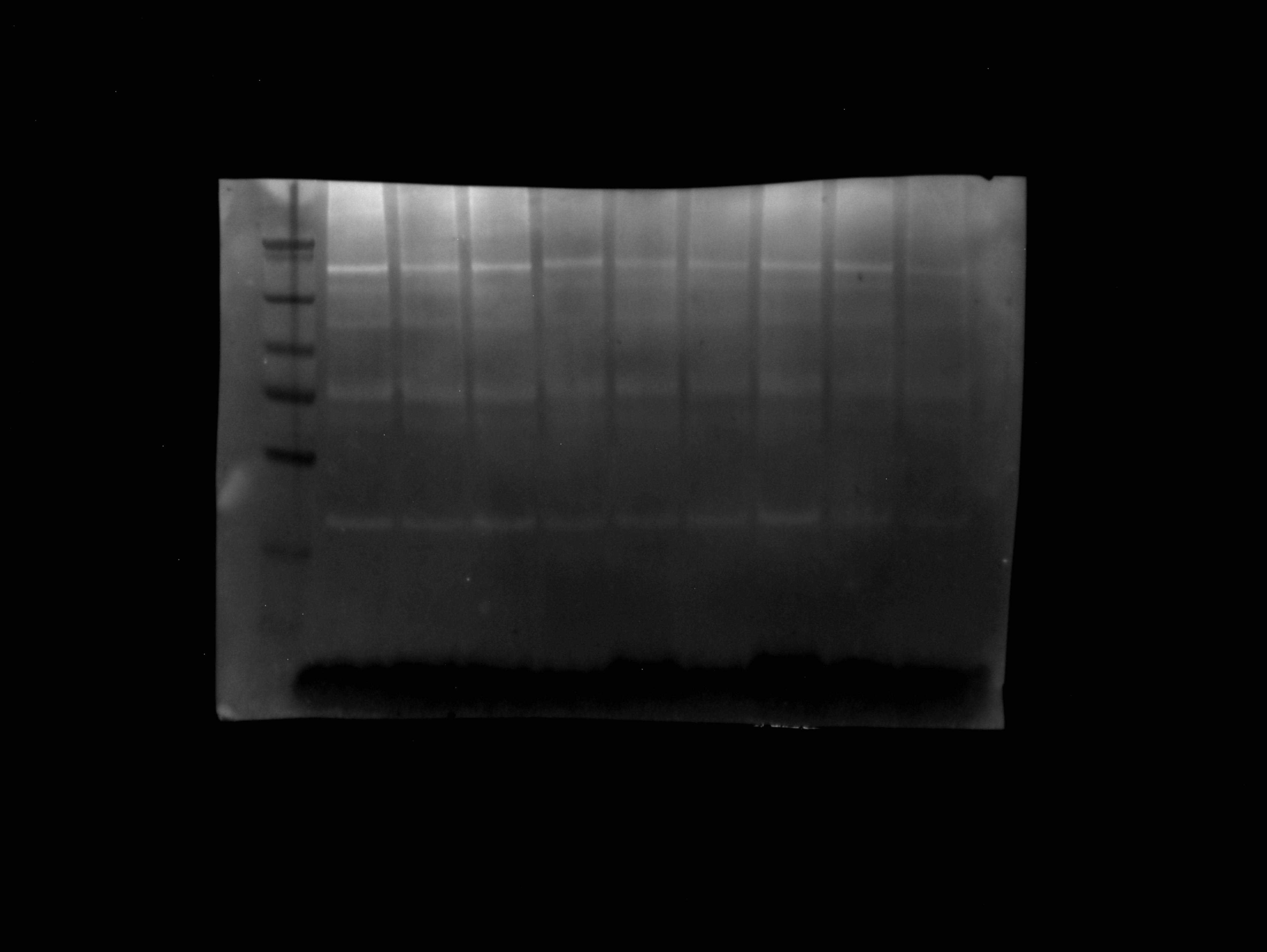

Supplement: Supplemental Information 5 [file peerj-12-17931-s005.jpg]
